# Editorial for the Special Issue “Potentially Toxic Elements Pollution in Urban and Suburban Environments”

**DOI:** 10.3390/toxics10120775

**Published:** 2022-12-11

**Authors:** Ilaria Guagliardi

**Affiliations:** National Research Council of Italy-Institute for Agricultural and Forest Systems in Mediterranean (CNR-ISAFOM), Via Cavour 4/6, 87036 Rende, Italy; ilaria.guagliardi@isafom.cnr.it; Tel.: +39-0984841427

Pollution by potentially toxic elements (PTEs) is becoming a serious and widespread issue in all environmental matrices because of accelerated population growth rate, rapid industrialization and urbanization, and other changes which have occurred in most parts of the world in the last few decades [1,2,3]. The increasingly worrying concern about the presence of PTEs in the environment has attracted considerable attention due to their potential impacts on ecosystem functioning and on public health because of their persistence and biotoxicity [4,5]. PTEs can in fact be transferred into the human body as a consequence of dermal contact, inhalation, and ingestion through the food chain and drinking water [6]. Unfortunately, PTEs are ubiquitous in all environmental compartments, and they have been widely detected worldwide [7,8]. In this context, environmental geochemistry and related subjects are elected matters to investigate, characterize, and reveal the patterns of inorganic elements together with geostatistical computations that are used to identify source patterns of different pollutants related to underlying geological features and/or anthropogenic activities [9,10].

The collection of the research carried out worldwide in this Special Issue “Potentially Toxic Elements Pollution in Urban and Suburban Environments” represents the need of the scientific community to characterize the behavior, transport, fate, and ecotoxicological state of PTEs in environmental matrices in both urban and suburban settings. The published research articles include, in fact, case studies from China to South Africa to Europe and interest various environmental compartments. Tomczyk-Wydrych et al. [11], Famuyiwa et al. [12], and Guagliardi et al. [13] approached, in their researches, the analysis of PTEs pollution in the soil matrix. Particularly, Tomczyk-Wydrych et al. [11] assessed the PTEs contamination of the topsoil in the selected part of the Zduńska Wola Karsznice railroad, in central Poland, and individuated the anthropogenic origin of the high level of copper, cadmium, lead, and others in the surface layer of soil, the risk to living organisms, and the destabilizing effect on ecosystems. The high accumulation index of copper, cadmium, and lead in the surface layer of soil indicated their anthropogenic origin.

Famuyiwa et al. [12] determined the concentrations and potential mobilities of Cr, Cu, Fe, Mn, Ni, Pb, and Zn in soils from public-access areas across the Lagos metropolis (Nigeria) and evaluated the risks associated with human exposure to PTEs, evidencing how the PTE levels are increasing due to rapid urbanization and industrialization. Concentrations of Cr, Cu, Fe, Mn, Ni, Pb, and Zn varied markedly in soils obtained from public-access areas across the megacity of Lagos. The calculations approached in the article also indicated the presence of non-carcinogenic risk for children, as well as carcinogenic risk for both children and adults.

Guagliardi et al. [13] individuated different types of pollution fronts, controlling for a structure of monitoring data sets in an area of Southern Italy, through one of the most powerful techniques for this purpose, the Self-Organizing Maps (SOMs) of Kohonen [14], identifying high-risk areas that can be targeted for environmental risks and public health. A combination of the analysis of major metals, minor metals, and PTEs, with the statistical treatment of SOMs, showed the geolithological formations and anthropogenic pressure on the territory. Anomalies ascribable to anthropogenic input in urban soils, referring to elements such as Pb and Zn, and some geogenic anomalous high values of As, Cr, and V mainly identified in peri-urban areas, were recognized.

For water compartments, both natural spring water and industrial wastewater were studied. More precisely, Infusino et al. [15] classified the spring water types of the Sila Massif (Calabria, Southern Italy) according to their hydrogeochemical features, identifying the factors controlling mineral waters using spatial variables focused on lithological settings and determining the vulnerability to nitrate occurrence in the spring waters. The 59 springs analyzed in this study showed that they were strongly affected by the geological nature of the rocks of the aquifers (granites, magmatites, acid granulites, biotic gneiss, etc.). Indeed, the waters are generally poor in mineral salts (very-low-mineral-content waters) with low calcium and magnesium content (soft).

Parrone et al. [16] analyzed field and laboratory data in order to evaluate the significant connections between aquifer mineralogy and groundwater geochemistry, with particular reference to As, and also to deepen its relationships with other PTEs in groundwater in the Sabatini volcanic apparatus, a volcanic–sedimentary aquifer in Rome, Italy. In this survey, the joint analysis of field data and laboratory observations suggested the existence of a diffused As geochemical background due to the water–rock interactions. This study pointed out that in volcanic areas, although the reductive dissolution of Fe oxyhydroxides could cause an enhanced localized release of As into groundwater, the anion exchange induced by specific exchangers (e.g., phosphates) with As adsorbed on the surface seems to be the most widely diffused process.

Pei and Sun [17] approached the problems of molybdenum pollution and difficult extraction and recovery in industrial wastewater in China, which has the largest reserves of molybdenum in the world. In this study, the influencing factors on the migration characteristics of Mo(VI) from the simulated trade effluent was examined using a newly designed membrane chemical reactor with mixed organic–water solvent (MCR-OW). The results showed that the MCR-OW was able to improve the efficiency of Mo(VI) migration from the simulated trade effluent, using the mixed diesel and NaOH (stripping solution) with the carrier of N-503 as renewal solution.

More attention was placed on an environmental compartment considered a lesser-known air pollutant such as dust, both in a road and a household. In fact, Mugudamani et al. [18] determined the influence of urban informal settlements on trace element accumulation in road dust from Ekurhuleni Metropolitan Municipality, South Africa, and their health implications. The outcomes of this study revealed that informal settlement activities have considerable influences on the accumulation of trace elements in road dust during the winter season. Major elements (SiO_2_ and P_2_O_5_) and trace elements (Cr, Cu, Zn, Zr, Ba, and Pb) were health concerns as they were above their corresponding average shale values.

In addition, Xiao et al. [19] collected road dust samples in Jiayuguan, Hexi Corridor, China and measured the concentration of PTEs to determine the pollution levels. They calculated the enrichment factor and geoaccumulation index of PTEs, identified their sources through principal component analysis, and assessed their health risks using the EPA health risk assessment model. Among the 12 PTEs (V, Cr, Mn, Co, Ni, Cu, Zn, As, Se, Cd, Ba, and Pb) in the road dust samples, the highest concentrations were for Mn, Ba, Zn, and Cr, and the concentration of PTEs in industrial areas was higher than the other three functional areas as a whole. The calculation of the Igeo showed that for most elements, the pollution level of the industrial area was higher than that of the rest of the areas. According to the source apportionment results, the PTEs in Jiayuguan road dust mainly came from geogenic–industrial sources, coal combustion, traffic sources, and oil combustion.

Jeong and Ra [20] evaluated PTE contamination and health risks posed by fine road dust (10–63 μm, <10 μm) collected in Apia City, Samoa. Cr, Co, and Ni concentrations in road dust with a particle size of 10–63 μm were higher than in fine road dust (<10 μm), while Cu, Zn, As, Cd, Sb, Pb, and Hg were present at relatively high concentrations in fine road dust. The PTE concentrations in road dust suggested that anthropogenic sources related to traffic activity rather than industrial emissions affect road dust in the study area. The results of correlation analyses and pollution assessments showed that only Cr, Co, and Ni were greatly affected by natural sources due to the weathering of volcanic parent rocks. In addition, relatively high concentrations were observed in sampling sites with high traffic volume, suggesting that contamination with these PTEs (Cu, Zn, Sb, and Pb) was mainly due to traffic activity. The ingestion exposure route posed a greater health risk than inhalation and dermal contact.

Gad et al. [21] detected the PTE levels in household dust and identified their spatial distribution in Cairo City, individuating the possible sources of PTEs in household dust using multivariate statistical analysis and assessing the potential health risk for children and adults’ exposure to PTEs. Their findings revealed that the levels of As, Cd, Cr, Cu, Hg, Mo, Ni, Pb, and Zn surpassed the background values of UCC, indicating anthropogenic influences; New Cairo recorded the slightest degree of contamination, ranging from considerably to very high pollution, while in other Cairo regions, household dust is very highly polluted. Elevated PTE concentrations in Cairo’s household dust may be due to industrial activities and heavy traffic emissions; the health risk assessment model showed that the vital route of potential PTE exposure that leads to both noncarcinogenic and carcinogenic risks is ingestion, followed by dermal and inhalation pathways. The noncarcinogenic risk was generally in the safe range for adults’ exposure. Children are at risk in some sites.

Li et al. [22] measured the concentrations of heavy metals (including As, Cd, Co, Cr, Cu, Ni, Pb, and Zn) in PM_2.5_ in Yuci College Town, Shanxi, China, for analyzing the source apportionment and assessing the health risks (noncarcinogenic and carcinogenic) of exposure to eight heavy metals, via inhalation exposure, during winter haze periods. Their results indicate that a total of 18% and 100% of the concentrations assessed in the samples for As and Cr, respectively, exceeded the corresponding threshold values of the Chinese Ambient Air Quality Standards and the WHO Global Air Quality Guidelines; the levels of As and Cr (VI) were also higher than those in some areas worldwide. Combustion was the largest contributor to PM_2.5_-bound heavy metals (37.91%), followed by traffic emissions (32.19%) and industrial sources (29.9%).

Finally, Wan et al. [23] studied the discharge patterns of PTEs from coking plants and their relationship with soil PTE contents in the Beijing–Tianjin–Hebei (BTH) region, China. This discharge of PTEs from the coking industry was found to be closely related to the accumulation of PTEs in soil. The discharge of Hg, As, and Cd from coking in the BTH region were relatively higher than those of other metals, which resulted in higher ecological risks of Hg, As, and Cd in this area. As result, scenario analysis also showed that the level of ecological risks would increase rapidly with an extending production time, especially for As, Cr, Ni, and Pb.

The published research articles are all of importance to face PTEs pollution in the environment through in-depth knowledge of the mechanisms that underlie the occurrence, distribution, migration, evolution over time of these pollutants and their effects on health and environmental risks.

Many challenges remain for researchers in environmental sciences, from the assessment of pollutants in all possible compartments, obtaining a harmonization in terms of sampling procedures, matrix characterization, preservation procedures, analytical methods, etc., to the remediation technologies involved in pollutant removal from matrices in contaminated sites, and building awareness for sustainable management of the territories which limit the pollution of environmental matrices.

According to the important results achieved (12 published papers and numerous ones that unfortunately have not passed scientific quality standards after careful peer review processes), my role as Guest Editor of this Special Issue was exciting and stimulating. The success of this Special Issue has really been a motivation and inspiration for me to continue the research in this fundamental topic for the protection of public health and ecosystems trying also to support the decision makers in the field of sustainable development implementation.

I would like to express my appreciation to all the authors for submitting their original contributions to this Special Issue and to reviewers for their essential part in assessing the suitability of the manuscripts and improving their quality. It would not be appropriate if I failed to mention the editors of Toxics for their kind invitation, and in particular Selena Li of the *Toxics* Editorial Office for her precious and tireless support.
